# An Affordable Image-Analysis Platform to Accelerate Stomatal Phenotyping During Microscopic Observation

**DOI:** 10.3389/fpls.2021.715309

**Published:** 2021-07-29

**Authors:** Yosuke Toda, Toshiaki Tameshige, Masakazu Tomiyama, Toshinori Kinoshita, Kentaro K. Shimizu

**Affiliations:** ^1^Japan Science and Technology Agency, Saitama, Japan; ^2^Phytometrics co., ltd., Shizuoka, Japan; ^3^Institute of Transformative Bio-Molecules (WPI-ITbM), Nagoya University, Nagoya, Japan; ^4^Kihara Institute for Biological Research, Yokohama City University, Yokohama, Japan; ^5^Department of Biology, Faculty of Science, Niigata University, Niigata, Japan; ^6^Department of Evolutionary Biology and Environmental Studies, University of Zurich, Zurich, Switzerland

**Keywords:** affordable phenotyping, real-time image analysis, stomatal density, stomatal size, microscopy

## Abstract

Recent technical advances in the computer-vision domain have facilitated the development of various methods for achieving image-based quantification of stomata-related traits. However, the installation cost of such a system and the difficulties of operating it on-site have been hurdles for experimental biologists. Here, we present a platform that allows real-time stomata detection during microscopic observation. The proposed system consists of a deep neural network model-based stomata detector and an upright microscope connected to a USB camera and a graphics processing unit (GPU)-supported single-board computer. All the hardware components are commercially available at common electronic commerce stores at a reasonable price. Moreover, the machine-learning model is prepared based on freely available cloud services. This approach allows users to set up a phenotyping platform at low cost. As a proof of concept, we trained our model to detect dumbbell-shaped stomata from wheat leaf imprints. Using this platform, we collected a comprehensive range of stomatal phenotypes from wheat leaves. We confirmed notable differences in stomatal density (*SD*) between adaxial and abaxial surfaces and in stomatal size (*SS*) between wheat-related species of different ploidy. Utilizing such a platform is expected to accelerate research that involves all aspects of stomata phenotyping.

## Introduction

Stomata are pores of plant leaves that regulate gas exchange. Plants modulate the degree of stomatal opening (aperture) to adjust CO_2_ uptake and water loss in response to environmental conditions such as light intensity, humidity, temperature and CO_2_ concentrations. In addition to the regulation of stomatal aperture, stomatal density (*SD*) and stomatal size (*SS*) are also known to influence gas exchange efficiency (Bertolino et al., 2019). From this viewpoint, quantification of such traits is important to gain insights into the molecular mechanisms that underlie the environmental adaptability of the plant. Genetic approaches such as Quantitative Trait Loci analysis and Genome-Wide Association Studies, in addition to forward chemical genetic screening, have successfully identified factors involved in such traits (Ziadi et al., 2017; Chen et al., 2020). To detect genetic and environmental effects on a quantitative trait, it is crucial to measure the data variation using a large sample (Tsuchimatsu et al., 2020). However, quantification of stomata-related phenotypes has often had to rely on manual methods, limiting the throughput of the research.

Recent technological advances in the computer-vision domain have allowed the development of various algorithms, pipelines or platforms to quantify stomata-related phenotypes through analysis of microscopic images. For example, Jayakody et al. (2017) have built a HOG classifier to detect the stomata of grapevine leaf imprints. Toda et al. (2018) have also utilized HOG in an image-analysis pipeline to detect the stomata of dayflower leaf disks. Meanwhile, Fetter et al. (2019) have utilized a convolutional neural network (CNN), a deep learning architecture, to identify stomata from a variety of microscopic images taken from various plant species. Other deep learning models, e.g., YOLO, SSD, and Mask R-CNN, have been proposed as useful adjuncts in stomata detection and trait measurement (Sakoda et al., 2019; Casado-García et al., 2020; Jayakody et al., 2021). As exemplified by those studies, deep learning has been demonstrated to be efficacious in the quantification of stomatal traits.

However, we faced several issues in implementing the above systems in a laboratory environment. First, to train a deep neural network model, a computer equipped with a high-performance graphics processing unit (GPU) and sufficient random-access memory (RAM) was required. Moreover, to use the trained deep learning model in daily analysis, an additional personal computer (PC), preferably also with a GPU, was ideally needed. This involves a high implementation cost to establish the image-analysis system. Second, we experienced difficulty in implementing real-time analysis that could observe and detect stomata on the fly. Attainment of such systems has been desirable for experimental biologists because they are expected to relieve the labor of injecting the acquired microscopic image into an independent program. However, manufacturers of laboratory-grade CCD/CMOS cameras designed for microscopic image acquisition often do not make drivers, software development kits or application programming interfaces available to users, but use proprietary dedicated software to run the devices. This makes it difficult for a “home-brewed” image-analysis program to access the camera connected to the microscope for on-site analysis.

To resolve such issues, we designed a microscopic system intended to analyze stomatal traits in real time, which can be easily and affordably prepared. The hardware system consists of an upright microscope connected to a USB video device class (UVC)-compatible camera and a Jetson Nano, a GPU-supported single-board computer. We chose each component to be generally available at electronic commerce sites (e.g., Amazon, eBay) at an affordable price, so that the total cost does not exceed USD 1,000. Moreover, the UVC compatibility of the camera allows it to be controlled from open-source computer-vision libraries. Using this system, we built an analysis pipeline to detect the stomata of wheat leaf blade imprints using an SSD, a deep learning architecture designed for object detection (Liu et al., 2015). The train/test dataset annotation and the model training were performed using free cloud services, which also minimized the preparation cost.

Using this platform, we investigated the phenotypic traits of wheat stomata. Here, we demonstrate that our platform can easily quantify wheat *SD* and *SS* in large numbers of samples. By increasing the sample number, we were able to detect the difference in the *SD* between adaxial and abaxial surfaces with high statistical confidence. Notably, a negative correlation between *SD* and *SS* within a single leaf was also detected. As exemplified by the case study, utilizing such a platform is expected to accelerate research involving all aspects of stomata phenotyping in fields such as plant physiology, breeding and agriculture.

## Materials and Equipment

### Required Hardware

#### Server PC

• NVIDIA Jetson Nano B01 (NVIDIA, United States).
• 5 V/4 A AC/DC power supply for Jetson Nano.
• UHS-I microSD card (preferably larger than 64 GB).
• USB A-MicroB conversion cable.
• LAN cable.
• Internet accessible environment.
• (Optional) USB memory for storing the acquired image.
• An additional PC or an adapter that can read/write a SD card image for the Server PC.

#### Microscope

• Upright C-mount trinocular microscope with ×4 and ×10 lenses, e.g., SW380T 40–2500X (Swift Optical Instruments, United States). Alternatively, a binocular microscope can be used with an additional eyepiece C-mount adapter.
• UVC-compatible with C/CS mountable camera with a resolution preferably over 8 MP. e.g., WE3170 (GAZO, Japan), ELP-USB13M02-MFV (ELP, China).

#### Client PC

• Arbitrary PC with USB 2.0 connection available.

### Required Software

#### Server PC

• Configuration provided by NVIDIA^1^. However, for the initial SD card image, we strongly prefer using JetCard,^2^ an AI development friendly configuration. This image enables users to skip the time consuming and complex installation of the python-related libraries including ones that are required by our GUI system, as well as Jupyter Notebook (Lab) Server and initial user account setup. Instructions written in the section “Methods” assume that users have used the JetCard image.
• We prepared a simple browser-based GUI that can be run in Jupyter Notebook^3^. This package also contains the stomata detection SSD model weights prepared as described in the Methods section. Alternatively, users can run their custom python code that receives camera input and simultaneously processes data.
• Users may connect the display to the Jetson Nano while setup, however, upon running the analysis program, the display must be disconnected and be controlled by the client PC (headless mode) to ensure sufficient free GPU memory for executing the deep neural network model and image analysis upon real-time analysis.

#### Client PC

• Any software that can perform an SSH connection to the server PC.
• Web browser.
• 1x USB-A port.
• The present system has been tested in the following PC environments, although most of the commercially available PC environments are expected to work. If the OS does not have an SSH client at default, download any arbitrary SSH client.

– macOS Catalina 10.15.7 with Google Chrome (91.0.4472.114).
– Windows 10 Pro version 1909 (OS Build: 18363.1440) with Microsoft Edge (91.0.864.59).

## Methods

### Configuration

#### Microscopy

• Remove the lens unit of the camera if present and mount it on the C-mount trinocular tube of the microscope. The camera can alternatively be mounted to the eyepiece of the microscope using the eyepiece/C-mount adapter.

#### Server PC

• Create the SD card image of Jetson Nano using the JetCard image at any available PC by downloading and writing the image file available at https://github.com/NVIDIA-AI-IOT/jetcard. Users will need a PC with an adapter to read/write SD card. Detailed instructions of how to prepare are thoroughly described at the website.
• Insert the SD card with its image into the Jetson Nano.
• Power on the Jetson Nano with headless mode (no monitor connected).
• Connect the microscopy camera to the USB A port of Jetson Nano.
• Connect the LAN cable to Jetson Nano.

– For security reasons, we do not recommend the Jetson Nano to be always connected to the internet. We prefer the LAN cable to be disconnected after the initialization step described below. After setup, the system can be run completely offline.

#### Client PC

• Initialization.

– Connect the client PC to the Jetson Nano with the USB A-Micro B cable.
– Open the terminal and establish SSH connection with the following command.

– ssh -p jetson jetson@192.168.55.1.
– Using the Jetson Nano as the server PC through USB connection will automatically assign its IP address to 192.168.55.1, which is the default value configured in NVIDIA Jetson series (e.g., Nano, Xavier, TX2) as of June 2021.

– Install additional python libraries with the following command in the terminal.

– pip3 install ipywidgets scikit-image.

• Download the GUI and dependencies with the following command. Ensure the user is in the home directory (e.g. cd $HOME).

– git clone https://github.com/totti0223/onsite_stomata_platform.git.
– cd onsite_stomata_platform.
– git clone https://github.com/tensorflow/models.git.

– Close the terminal.
– Disconnect LAN cable from the Jetson NANO.

• GUI execution for stomatal detection.

– Open the terminal again and establish SSH connection this time with port forwarding.

– ssh -p jetson -L 8888:localhost:8888 jetson@192.168.55.1.

– If the JetCard image is used for Jetson Nano, port 8888 is occupied by JupyterLab instead of Jupyter Notebook. Both applications are compatible at the current state.

– Open a web browser, and type the following url for JupyterLab connection.

– localhost:8888.

– Type jetson for password.
– Locate and click the folder “onsite_stomata_platform” to move to its directory.
– Click and open “main_En.ipynb.”
– Execute each cell from the top. Execute the cell as described below and the GUI will start inside the notebook.

– _ = pme.stream(pipeline_func = pipeline_func, output_directory = None, camera_id = 0, videocapture_api_backend = 200, camera_initial_settings = {‘format’: [‘M’, ‘J’, ‘P’, ‘G’], ‘height’: 768, ‘width’: 1024, ‘fps’: 30}).

– The above code assumes users are using the ELP-USB13M02-MFV camera. If users want to test their Jetson Nano with a USB Web Camera or other input devices, simply delete camera_initial_settings = {…} from above.
– If the GUI would not start or execute properly due to JupyterLab specific configuration or version incompatibility, shutdown the ipynb notebook and reaccess by Jupyter Notebook. In brief, change the “/lab” to “/tree” in the URL. See https://jupyterlab.readthedocs.io/en/stable/getting_started/starting.html for details.

• GUI execution for custom image analysis interface.

– If users want to use their own stomata detection model in their GUI, the most simple way is to rename the existing “saved_model” folder to “_saved_model” and copy their own “saved_model” folder into the directory. Depending on their training condition and *SS*, users may have to change their input image size from the camera (from the GUI pulldown menu) to obtain optimal results. Notably, if users would like to prepare an image analysis module other than bounding box detection (e.g., instance segmentation), the user will need to prepare a custom pipeline func. In any case, the module can be modified in the notebook cell, in which the existing codes are self explanatory for users who have sufficient skills to prepare their own custom pipeline.

• Access https://github.com/totti0223/onsite_stomata_platform for further details online and future updates.

### Generation of Stomata Detection Model

For annotating images used for machine-learning model training, we used Labelbox^4^, a cloud labeling service that is currently free for academic usage. We uploaded and labeled 697 wheat leaf imprint images so that each stoma for the training set was annotated with a circumscribed bounding box. Each image had a resolution of 2048 × 3072 (height × width) pixels that were acquired as described in the following section “Plant Materials, Growth Condition, and Sample Preparation.” Annotated dataset was then exported from Labelbox to the local environment in JSON format. Next, labeled images were resized to 1024 × 1536, padded with black pixels to the size of 1024 × 2048, and were split into two sized 1024 × 1024. The image transformation and the converting bounding-box coordinates were performed using Albumentations (Buslaev et al., 2020), an image augmentation library. Sets of images and annotations were then converted to COCO json format, then finally to TFRecord format.

The training of the stomata detection model was performed by following the steps of the section “Training and Evaluation with TensorFlow 2” of the Tensorflow Model Garden repository^5^. The training process was run in Google Colaboratory^6^, a freely available cloud programming service with GPU accessibility. Detailed codes and instructions to reproduce the regarding system as well as the stomata detection model is described in Google Colaboratory executable notebook^7^ hosted at https://github.com/totti0223/onsite_stomata_platform. Briefly, the default config parameters provided in the repository were used for training. We used an SSD (Liu et al., 2015) with MobileNetV2 backbone, pretrained with COCO dataset, with an input size of 640 × 640 (Refer to ssd_mobilenet_v2_fpnlite_640 × 640_coco17_tpu-8 at Tensorflow Model Garden). As a result, we obtained a model that detects stomata with a mean Average Precision (mAP) of 0.825 and 0.636 with the intersection-over-union (IoU) threshold of 0.3 and 0.5 against the test dataset, respectively. The trained model was downloaded from Google Colaboratory to the local environment as a SavedModel format, and used for the GUI. Notably, the test dataset was created by acquiring 50 images using the microscopy proposed in this research with a resolution of 768 × 1024.

### Plant Materials, Growth Condition and Sample Preparation

#### Training Data

A series of 25 bread wheat accessions with serial numbers from LPGKU2305 to LPGKU2329 (National BioResource Project–Wheat, Japan) and a Swiss winter wheat cultivar “ArinaLrFor” (Singla et al., 2017) were used as training data. The first leaves of young plants and the flag leaves after heading were collected from those wheat accessions in the greenhouse. The epidermal thin imprints were prepared by pasting and drying clear nail polish on the leaves. In some cases, first imprints were taken from the leaves using dental impression material (Dent Silicone AQUA, Shofu Inc., Kyoto, Japan) and used as the templates for the nail-polish imprints. The nail-polish imprints were put on glass slides and observed directly or after mounting with glycerol. Of note, samples were placed on the microscopic stage so that stomata were aligned as horizontally as possible. The microscope was a normal bright-field microscope, Olympus BX61 (Tokyo, Japan), equipped with a normal lens UPlanFLN. The camera was an RGB camera of 6.3 MP CMOS, a WRAYCAM-NOA 630 (Wraymer Inc. Osaka, Japan). These images were used for initial training for the detection model described in the prior “Generation of Stomata Detection Model” section. Other bread wheats, a cultivar Yumechikara and a synthetic hexaploid wheat, were used to acquire datasets for validation. These samples were observed using the same method as the samples used for the measurements below.

#### *SD* and *SS* Measurements

A hexaploid bread wheat *Triticum aestivum* (cultivar: Chinese Spring), a tetraploid wheat *Triticum turgidum* (extracted tetraploid wheat harboring AABB subgenomes of Chinese Spring) (Yang et al., 1999), diploid wheat-relative grasses *Triticum urartu* (accession: KU-199-01) and *Aegilops tauschii* (accession: KU-2076), and a model grass *Brachypodium distachyon* (accession: Bd21) were used in this study. The seeds were germinated on moist filter papers in the dark at 4°C, then the seedlings were grown in plant pots under continuous white LED light in an air-conditioned room maintained at 22°C. The first leaves of four-leaf-stage plants were used for observation. Microscopic images were taken from three plants of each species. The slides were prepared using the same method as for preparing the training data described above. The microscope, camera and image processing devices were as described above.

To sample images from a wide range of leaf areas in an unbiased manner, images were acquired according to the following protocol. At first, we decided a whole target area to be acquired and prepared the imprint. Approximately a middle third of total leaf length was selected as the target because the middle parts of wheat leaves show smaller variation in *SD* among subsamples than subsamples from the distal and proximal parts of the leaves (Teare et al., 1971). Second, the angles of the imprint and camera were adjusted to align cell files along the left-right axis in the image. Third, start acquiring a series of images from the top left and scanned across the target area to the bottom right. Each horizontal scan-lines is distant from the next ones with approximately the image size. When the current frame displayed is not good due to distortion, breaking, bubbles or shallowness of the imprint, we ignored that frame and went further to a better frame. During such a data quality evaluation, the real-time feedback was helpful to know whether or not the image quality was in a permissible range.

### Data Analysis and Visualization of *SD* and *SS*

To calculate the *SD*, the total number of automatically detected stomata was divided by the microscopic field area (0.984 mm^2^ in our hardware setting). To measure the *SS*, the *x*- and *y*-lengths of each bounding-box were used as stomatal length and width, respectively, after scaling the values from units of pixels to micrometers (1.116 μm/pixel for ×4 lens, and 0.445 μm/pixel for ×10 lens). The bounding-box size of the stomata may be incorrect when the box coordinates contain a lower limit (0) or upper limit (1024 for the *x*-axis and 768 for the *y*-axis) because some part of the stomata protrudes from the image. Therefore, these marginal stomata were ignored in the *SS* calculation. The ground truth of stomatal length and width were manually measured on imageJ^8^ with Rectangle selection tool. Data visualization by scatter, density, box and jitter plots was performed using the *ggplot2* package (Wickham, 2016) in the R language^9^. Statistical tests including *t* test and correlation tests were performed using the *stats* package in the R language.

## Results

### Platform Appearance

**FIGURE 1:**
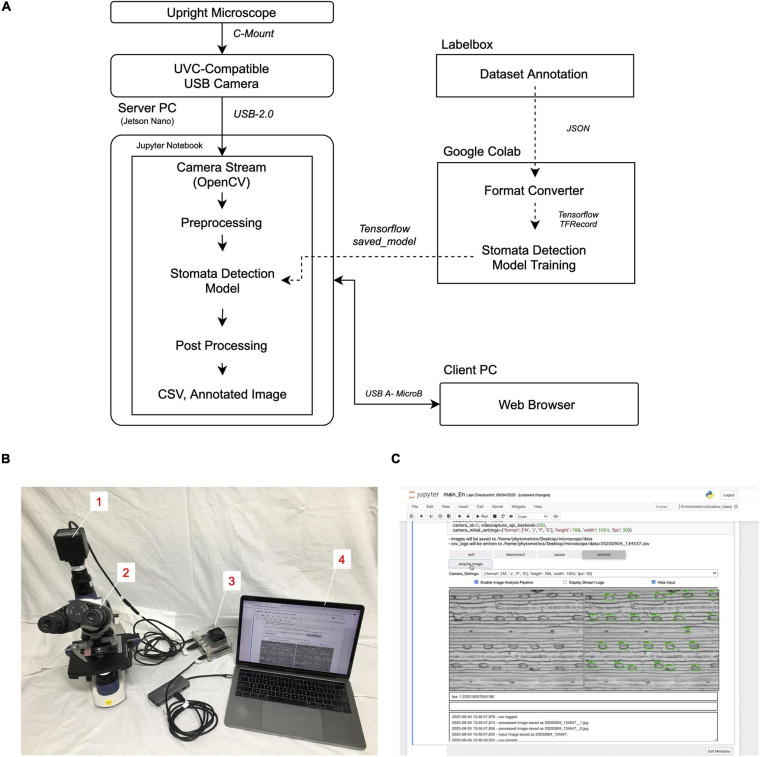
Stomatal detection platform. **(A)** Schematic diagram of the workflow. **(B)** Appearance of the platform. Numbers in insets describe the individual components. 1, UVC-compatible camera (ELP-USB13M02-MFV); 2, upright trinocular microscope (SW380T); 3, server PC (Jetson Nano B01); 4, client PC (Macbook Pro 13-inch, 2017). **(C)** Screenshot of the GUI run in the client PC through the Google Chrome web browser.

The appearance of the platform and the schematic diagram of the dataflow prepared by following the Methods section are shown in Figures 1A,B. The camera input image was processed in the server PC (Jetson Nano), while the client PC was connected to the server PC by a web browser through a USB connection. Therefore, the latter does not require a specific operating system or hardware specification, which allows multiple users to connect their PCs to the platform without installing any specific programs (Supplementary Movie)^10^.

The executed GUI is embedded inside the Jupyter Notebook (Figure 1C and Supplementary Figure 1). Moreover, the raw program code of the image-analysis module is written directly inside the cell of the Notebook. This allows easy program development and debugging for any users upon customization. In our configuration, the camera image sequence is processed through an SSD to obtain the coordinates of the detected stomata and then displayed as an annotated output. The processing speed of the platform depends on the camera input image size and the content of the image-analysis program, as well as the presence or absence of a display of the annotated image. In our configuration, it was about 1.4 frames per second.

### Stomatal Density Measurement

**FIGURE 2:**
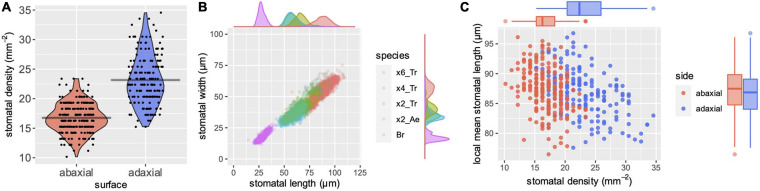
A case study of *SD* and *SS*. **(A)** Automatically measured *SD* of abaxial (red) and adaxial (blue) surfaces. The first leaves from bread wheat (*Triticum aestivum*) were observed at the four-leaf stage of seedlings. Gray horizontal bars indicate mean values. **(B)** Automatically measured *SS* of different species with a series of genome sizes. Besides a scatter plot of length (*x*-axis) and width (*y*-axis), the density plots along each axis are shown. “x6_Tr,” “x4_Tr,” “x2_Tr,” “x2_Ae,” and “Br” indicate hexaploid *T. aestivum*, tetraploid *Triticum turgidum*, diploid *Triticum urartu*, diploid *Aegilops tauschii* and diploid *Brachypodium distachyon*, respectively. More than a thousand stomata were measured for each species. Abaxial stomata from the former four species and adaxial stomata of *Brachypodium* were measured because the *Brachypodium* has rich abaxial prickle hairs that hinder automatic measurements. **(C)** Local *SD* and local mean stomatal length of bread wheat (*T. aestivum*). Data were collected by automatic measurements. Each point represents a single microscopic image with a size of 0.984 mm^2^. Besides a scatter plot of *SD* (*x*-axis) and length (*y*-axis), the box plots along each axis are shown.

To test the performance of stomata counting, we compared stomatal numbers per unit area (*SD*) from different types of tissues. In general, *SD*s differ between adaxial and abaxial leaf surfaces. In the case of Triticeae, the adaxial surface has a higher *SD* than the abaxial (Rajendra et al., 1978; Wang and Clarke, 1993). We measured *SD*s from adaxial and abaxial imprints of bread wheat leaves. More than 150 images, each of a 0.984 mm^2^ microscopic field, were analyzed. The mean *SD*s were 23.2 and 16.8 stomata/mm^2^ for adaxial and abaxial surfaces, respectively (Figure 2A). The true *SD*s, that is, the manually counted data, were 22.5 and 16.2 stomata/mm^2^, indicating error rates of only 2.84 and 3.70% for *SD* estimation.

Despite the obvious difference in *SD* between adaxial and abaxial surfaces as shown in Figure 2A, the stomatal counts of single images varied, and the distributions of adaxial and abaxial surfaces largely overlap. This means that no significant difference between the surfaces can be detected in some cases of small samples. When four images were randomly sampled from our dataset and Welch’s *t* tests were performed, 3,652 of 10,000 (∼37%) simulations resulted in no significant difference (*p* > 0.05). In the case of 10-each random samples, only 33 of 10,000 (0.33%) simulations resulted in no significant difference, confirming the importance of sample size in statistical tests. While these simulations were performed from data for manually counted *SD*, the same random sampling with our automatically estimated *SD*s that included some errors resulted in no significant difference in 4,907 (∼49%) and 81 (0.81%), respectively, of 10,000 simulations of 4- and 10-each random samples. These simulations suggest that increasing sample size has a higher impact than increasing accuracy in *SD* estimation and statistical power, given that the error rate is sufficiently low.

In addition, we asked whether our phenotyping system can also be used to quantify the stomatal patterning parameter. Our image acquisition protocol provides the images in which cell files are aligned horizontally, thus the y-coordinates of stomata are limited to the position of stomatal files. This property allows us to automatically collect the interval lengths between stomatal files along the *y*-axis of the image (Supplementary Figure 2A). Such analyses for the interval lengths were performed for our adaxial and abaxial datasets of the bread wheat. The results indicate that there are two types of intervals, namely around 130 and 230 μm, in the abaxial surface (Supplementary Figure 2B). On the other hand, the adaxial data show continuously varying interval lengths from 140 to 240 μm (Supplementary Figure 2C). As exemplified by those results, the stomatal position data obtained from the present system can be used to quantify some aspects of the stomatal pattern.

### Stomatal Size Measurement

Our analysis system detects each stoma as a bounding box and records each box size as representing the individual *SS*. Thus, the x-length and y-length indicate the stomatal length (guard cell length) and stomatal width (sum of guard cell widths, subsidiary cell widths and pore width), respectively, when the leaf distal–proximal axis corresponds to the left–right axis of the microscopic field (Supplementary Figure 3A). Comparison between the predicted and hand-measured *SS* of wheat-related species with different *SS* below resulted in Pearson’s correlation coefficients of *r* = 0.92 and *r* = 0.73 for stomatal length and width, respectively (Supplementary Figures 3B,C).

The mean *SS*, or the range of *SS*, is a species-specific trait, and useful for taxonomic classification in a case where no macroscopic trait is available to classify the different species clearly (Aryavand et al., 2003; Boza Espinoza et al., 2020). It is known that *SS*s correlate well with genome size in Triticum and other plants (Masterson, 1994; Beaulieu et al., 2008). Hexaploid bread wheat (*T. aestivum*) has a large genome consisting of A, B and D subgenomes (∼17 Gb) (International Wheat Genome Sequencing Consortium (IWGSC) et al., 2018). Tetraploid wheat with A and B subgenomes has approximately two-thirds of the hexaploid genome size. The diploid wild species, *A. tauschii* and *T. urartu*, which are the progenitors of bread wheat D and A subgenomes, respectively, have approximately a third of the genome size (Luo et al., 2017; Ling et al., 2018). We measured the *SS* of these plants using our bounding-box measurement system. The mean *SS*s were 87.2 μm × 54.4 μm, 66.3 μm × 40.7 μm, 59.0 μm × 36.4 μm, and 57.4 μm × 33.9 μm (length × width) for the AABBDD hexaploid, AABB tetraploid, AA diploid and DD diploid species, respectively, showing a clear correlation with the genome sizes (Figure 2B). This result shows that our automatic measurement performs well for a particular variation in *SS*, and is thus useful to describe this species-specific stomatal trait. Interestingly, the *SS* distributions partly overlapped between plants of different genome sizes, as shown by the scatter and the density plots (Figure 2B). In addition to determining the mean values, revealing such variations in a large sample number is important to identify the presence or absence of significant differences among species.

*Brachypodium distachyon*, a model grass, has a small genome (0.27 Gb) (The International Brachypodium Initiative, 2010). The molecular mechanism of formation of the dumbbell-shaped stomata of grass has been demonstrated in genetic studies using this species (Raissig et al., 2017). Its stomata are so small that only a portion of the stomata were detected when the low magnification (×4) lens was used, as was the case for wheat stomata in the present study (Supplementary Figure 4). This was because our model was trained with images of large wheat stomata. However, they were successfully detected with a lens of greater magnification (×10) (Supplementary Figure 4). The *Brachypodium* stomatal length and width were 27.9 ± 3.5 and 17.3 ± 2.8 μm (mean ± standard deviation), respectively (Figure 2B). Most cereal crops and wild grass species have genome sizes intermediate between those of *B. distachyon* and *T. aestivum*, thus their stomata can be expected to be detected if the image is acquired with an appropriate lens and resolution.

### Correlation Between *SD* and *SS*

Previous studies have demonstrated that the variation in *SS* within a species is negatively correlated with the *SD* of the tissues observed (Rajendra et al., 1978). Such a negative correlation has been reported between different genotypes and between different leaf positions in a shoot. However, our knowledge is very limited (Smith et al., 1989) about whether the same relationship exists between small microscopic fields within leaves of the same genotype, leaf position and growth condition. We found that this negative correlation was observed in our dataset that consisted only of the first leaves from a single cultivar (Figure 2C). The adaxial and abaxial datasets showed significant negative correlations (*p* = 6.26e–08 and 1.55e–07, respectively, in Pearson’s correlation test) between mean stomatal lengths and *SD*s of different microscopic fields. Interestingly, the mean stomatal lengths were almost the same for adaxial and abaxial surfaces (86.7 ± 3.9 and 87.2 ± 3.7 μm, respectively), although the *SD*s were different (Figure 2C). This may suggest that the size of each stoma is determined according to the local *SD* of only a small surrounding region, but the response to the local *SD* differs between adaxial and abaxial stomata. Our system and others that support high-throughput phenotyping can contribute to obtaining greater insight into such unknown developmental mechanisms.

## Discussion

In this study, we proposed a platform that enables real-time stomata detection using microscopic observation. The setup cost of the hardware does not exceed USD 1,000, and the stomata detection model and the training data labeling can be prepared based on freely available services. Using the platform, we demonstrated *SD*s and their variation in adaxial and abaxial leaf surfaces, and characterized the *SS* distribution in several wheat-related species of different genome sizes. In addition, we could show that the adaxial and abaxial stomata in a bread wheat exhibit the same mean size even though they show different densities, and *SD* and *SS* of each surface correlate negatively. Our results indicate that experimental biologists can benefit from these cutting-edge technologies in image processing, not only by developing the algorithm but also by using free cloud services and reasonably inexpensive hardware, implementing real-time image processing and a user-friendly user interface. We discuss below some possible options, applications and future challenges of the present system.

In recent years, many companies have released inexpensive single-board computers. Of these, we used the Jetson Nano for the system because at present it is one of the most affordable GPU-harboring PCs (approx. USD 60). However, the GPU memory of the Jetson Nano is limited to 2 GB, which restricts the deep learning architecture that can be loaded into the pipeline. Superior NVIDIA Jetson models such as XAVIER and the TX2 series possess larger GPU memory, thus providing more choices of system configuration.

For stomata detection, we utilized a deep learning-based object detection algorithm to infer the bounding-box coordinates of the stomata. Because the stomata are always oriented horizontally both in our dataset and under observation conditions (Figure 1C), the bounding-box coordinates obtained can be used to estimate the width and length of the stomata. Although further refinement of the model is potentially needed to improve accuracy, the current parameters were adequate to highlight the differences in *SS* between species (Figure 2B). Application of alternative deep learning architecture intended for segmentation, such as Mask R-CNN (He et al., 2017), is expected to achieve a more precise measurement of the morphology of the stomata (Jayakody et al., 2021).

We focused on the detection of stomata from wheat-related species, which have the dumbbell-shaped stomata typical of grass plants. Similarly, to the increasing reports of stomata detection of kidney shaped stomata of dicots, detection of dumbbell shaped stomata of grass are also starting to be established, exemplified by that of wheat (Sun et al., 2021). In the present study, the training dataset was prepared from only hexaploid wheat cultivars with some augmentation, thus the variations in *SS* and morphology of diploid and tetraploid species were not included. Despite this limitation, the trained model detected most of the stomata from diploid and tetraploid species. This possibly reflects the morphological similarity of dumbbell-shaped stomata in imprint images. Enhancing the generalization of performance to detect the stomata of various grass species might be accomplished with little effort.

Image processing methods may not perform well because of difficulties that are common in biological images (Uchida, 2013). Raw microscopic images acquired on-site often include low-quality data unsuitable for analysis (Koho et al., 2016), such as out-of-focus images, nonuniform lighting or physical damage of the sample. These are often not taken into consideration during the development of analysis algorithms, and can be a potential difficulty in operation. An advantage of real-time analysis is that we can prevent the acquisition of such low-quality images because we can judge the quality of image-analysis results during observation. In the case of stomatal detection using imprint images, causes for these may include contamination by dust or distortion or a shallow imprint. It is costly to use manual analysis or to develop another program to complement the data. Thus, real-time image analysis during observation enables quality assurance of the analyzed data by the operator. In addition, high-speed image analysis enables a time-course analysis, which reveals the dynamics of stomatal aperture when living cells are observed (Sun et al., 2021). There is another potential benefit of real-time image processing for the development of further automatic high-throughput phenotyping systems. Just as an automatic car-driving system regulates the speed in response to the real-time detection of pedestrians and road signs in the camera images, it is possible to move the stage automatically and acquire multiple images using a program that links the microscope camera and a motorized stage. The functionality of an imaging system with an automated motorized stage has been proposed for high-throughput stomatal phenotyping (Millstead et al., 2020). There are motorized stages that are commercially available at a low price and that are controllable from a PC. Real-time image processing systems can expand the possibilities of cooperation between computer and robot as well as cooperation between computer and human.

  While there are some potential challenges for further generalization of performance and higher throughput, our present approach will provide the possibility for many experimental biologists to introduce a cost-effective high-throughput system that will accelerate a range of studies involving stomata-related trait analysis.

## Data Availability Statement

The raw data supporting the conclusions of this article will be made available by the authors, without undue reservation.

## Author Contributions

YT and TT directed and designed the study. YT wrote the program codes and assembled the analysis platform. TT conducted the biological experiments, collected the wheat images, and involved in the conceptualization of this research. YT and MT annotated and verified the image dataset. KKS and TK provided advice to assure the scientific validity of the project. All of the coauthors were involved in the writing and verification of the manuscript.

## Conflict of Interest

YT and MT were employed in phytometrics, co., ltd. The remaining authors declare that the research was conducted in the absence of any commercial or financial relationships that could be construed as a potential conflict of interest.

## Publisher’s Note

All claims expressed in this article are solely those of the authors and do not necessarily represent those of their affiliated organizations, or those of the publisher, the editors and the reviewers. Any product that may be evaluated in this article, or claim that may be made by its manufacturer, is not guaranteed or endorsed by the publisher.

## Footnotes

1
https://developer.nvidia.com/embedded/learn/get-started-jetson-nano-devkit

2
https://github.com/NVIDIA-AI-IOT/jetcard

3
https://github.com/totti0223/onsite_stomata_platform

4
https://labelbox.com

5
https://github.com/tensorflow/models/blob/master/research/object_detection/g3doc/tf2_training_and_evaluation.md

6
https://colab.research.google.com

7
https://colab.research.google.com/github/totti0223/onsite_stomata_platform/blob/main/StomataDetectorModelTraining.ipynb

8
https://imagej.nih.gov/ij/

9
https://www.r-project.org/

10
https://youtu.be/CHk6Mw3kabc
