# Corrigendum: An Affordable Image-Analysis Platform to Accelerate Stomatal Phenotyping During Microscopic Observation

**DOI:** 10.3389/fpls.2021.793369

**Published:** 2021-11-03

**Authors:** Yosuke Toda, Toshiaki Tameshige, Masakazu Tomiyama, Toshinori Kinoshita, Kentaro K. Shimizu

**Affiliations:** ^1^Japan Science and Technology Agency, Saitama, Japan; ^2^Phytometrics co., ltd., Shizuoka, Japan; ^3^Institute of Transformative Bio-Molecules (WPI-ITbM), Nagoya University, Nagoya, Japan; ^4^Kihara Institute for Biological Research, Yokohama City University, Yokohama, Japan; ^5^Department of Biology, Faculty of Science, Niigata University, Niigata, Japan; ^6^Department of Evolutionary Biology and Environmental Studies, University of Zurich, Zurich, Switzerland

**Keywords:** affordable phenotyping, real-time image analysis, stomatal density, stomatal size, microscopy

In the original published version of this article, a note indicating that Yosuke Toda and Toshiaki Tameshige both contributed equally to this work and are share first authorship was omitted.

The authors apologize for this error and state that this does not change the scientific conclusions of the article in any way. The original article has been updated.

## Publisher's Note

All claims expressed in this article are solely those of the authors and do not necessarily represent those of their affiliated organizations, or those of the publisher, the editors and the reviewers. Any product that may be evaluated in this article, or claim that may be made by its manufacturer, is not guaranteed or endorsed by the publisher.

