# Supplementary figures and images for: An Affordable Image-Analysis Platform to Accelerate Stomatal Phenotyping During Microscopic Observation
Source: Front Plant Sci. 2021 Jul 29;12:715309. doi: 10.3389/fpls.2021.715309 (PMC8358771; doi:10.3389/fpls.2021.715309)

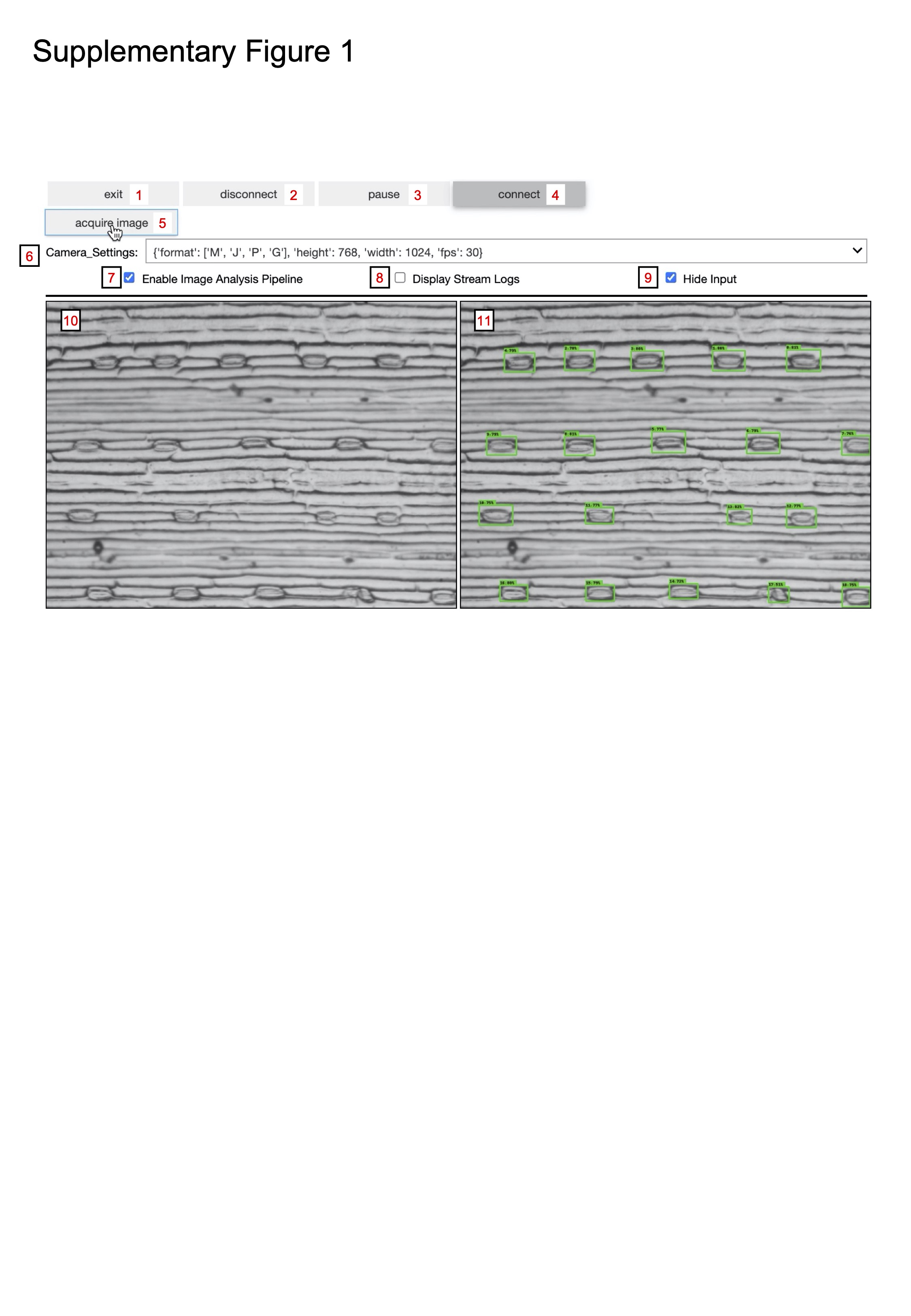

Supplement: Supplementary file 1 [file Image_1.JPEG]

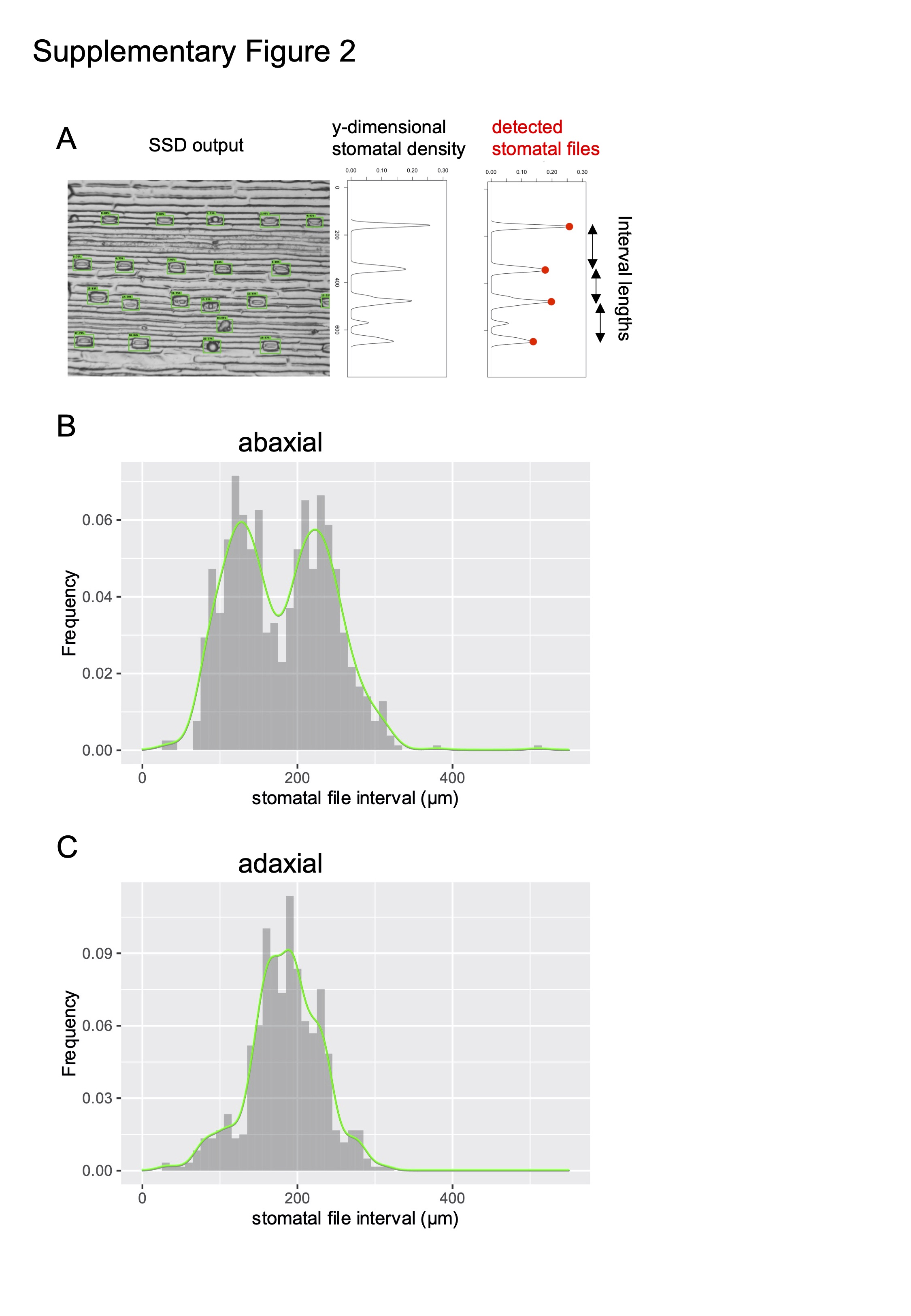

Supplement: Supplementary file 2 [file Image_2.JPEG]

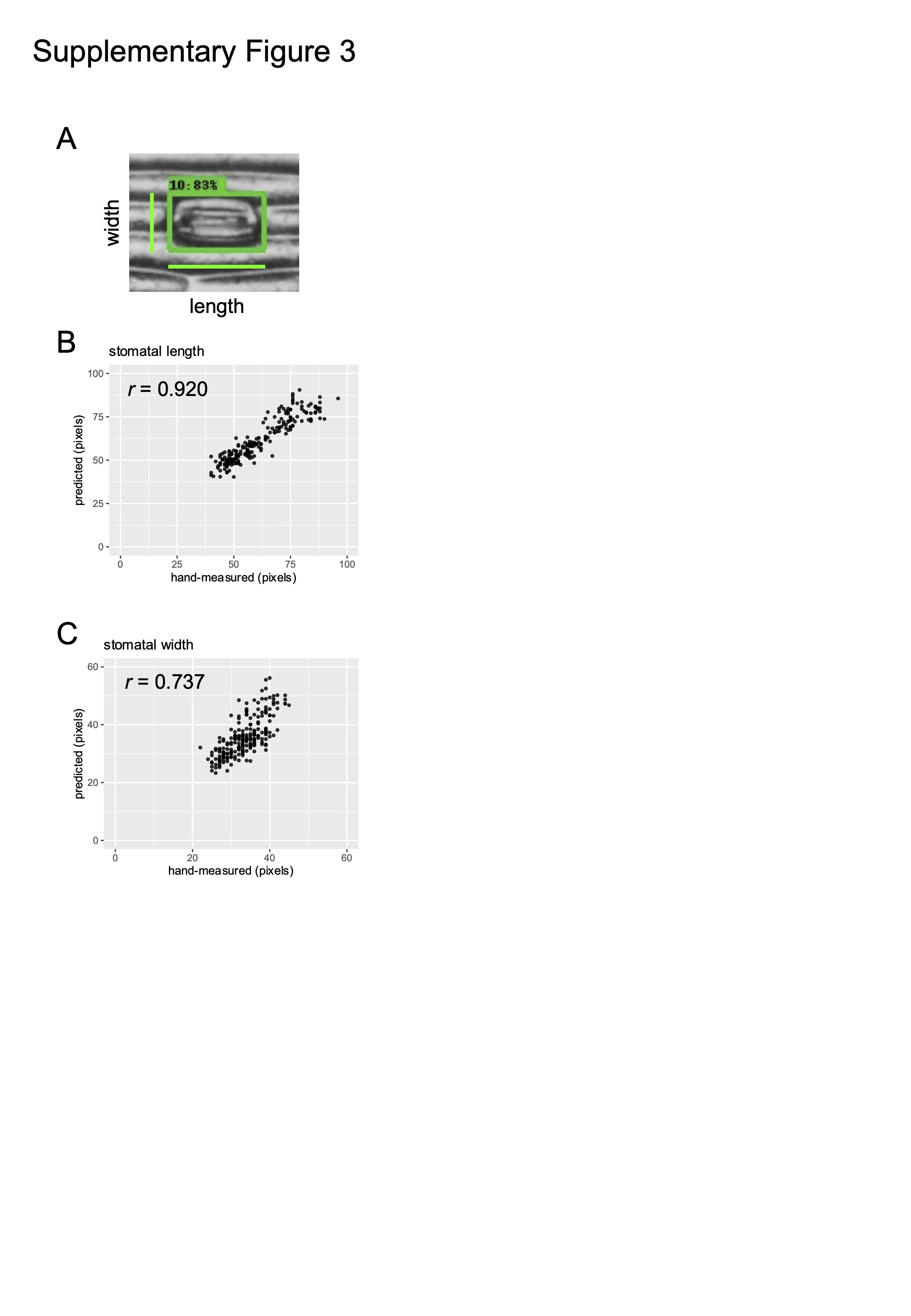

Supplement: Supplementary file 3 [file Image_3.JPEG]

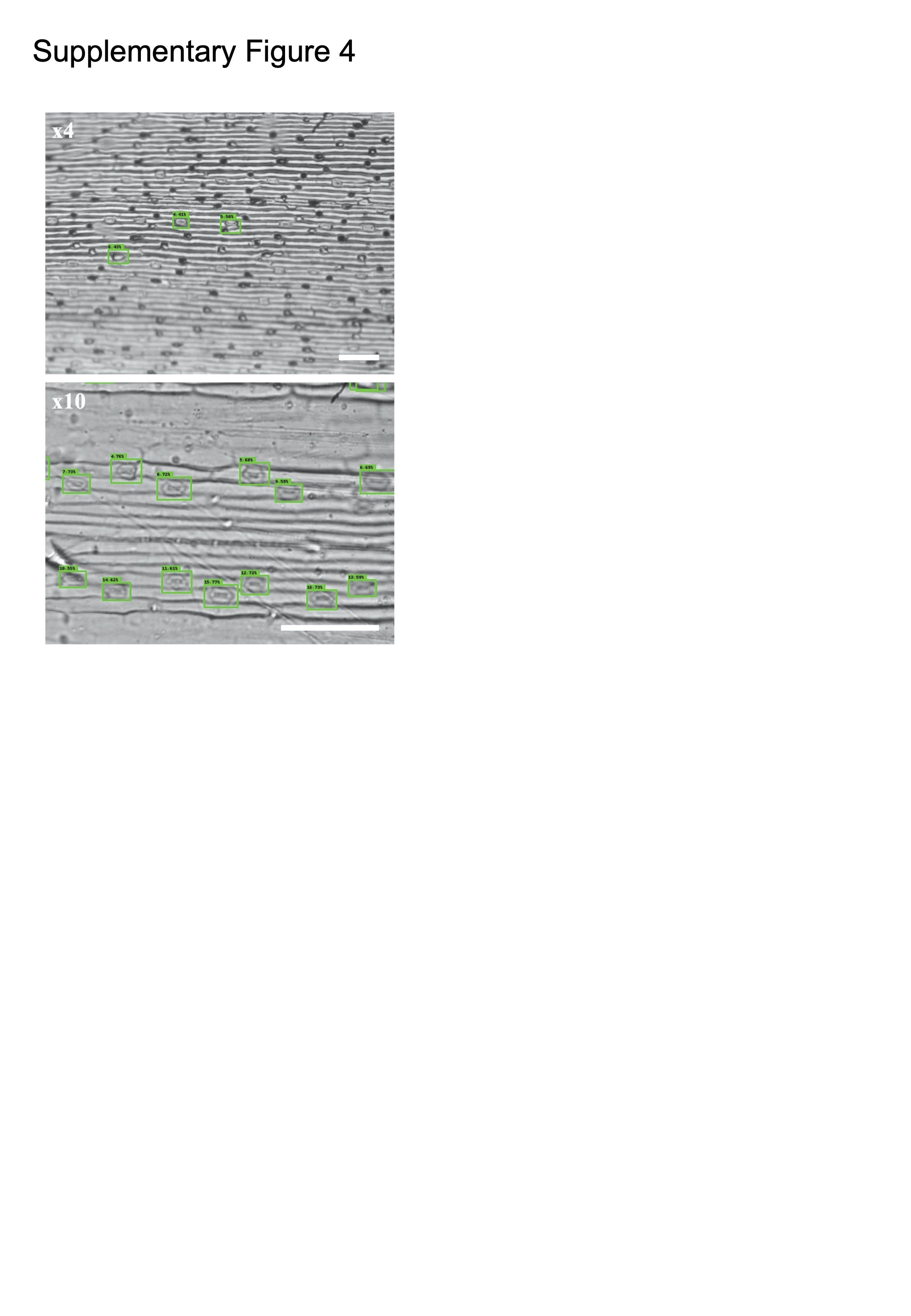

Supplement: Supplementary file 4 [file Image_4.JPEG]
