# Supplementary material for: An Affordable Image-Analysis Platform to Accelerate Stomatal Phenotyping During Microscopic Observation
Source: Front Plant Sci. 2021 Jul 29;12:715309. doi: 10.3389/fpls.2021.715309 (PMC8358771; doi:10.3389/fpls.2021.715309)
Supplement: Supplementary file 5 [file Data_Sheet_1.docx]

Supplementary Material

# Supplementary Data

# There is related information not included in the main text or figures. The source code and related files are on a github repository (<https://github.com/totti0223/onsite_stomata_platform>) as mentioned in the main text. The Supplementary movie is on YouTube (<https://youtu.be/CHk6Mw3kabc>) as mentioned in the main text.

# Supplementary Figure

We upload a Supplementary Figure as a JPG file. Please refer the legend below.

**Supplementary Figure 1. Close up view of the GUI of real-time analysis mode.**

(1) button “exit”: Finish the program. After this, the user can start another round of observation by running the relevant program again.

(2) button “disconnect”: Disconnect the camera. After this, the user can reconnect to the camera by clicking “Connect” and save data into the same directory and csv file.

(3) button “Pause”: Stop the input from the camera tentatively. When the user switches from “Connect” to “Pause”, camera will be still active, so can quickly reconnect to the camera by clicking “Connect”. However, if the user would like to do other tasks for a while, “disconnect” is recommended because “Pause” continues to make a certain load onto the computer.

(4) button “Connect”: Start connection to the usb camera. The live streaming images will be displayed on the window below.

(5) button “acquire image”: Save the current image. If it is “Pause” mode, the image being displayed will be saved. When “enable image analysis pipeline” (see below) is checked, the grayscale image (analysis input) and stomata-marked image (analysis output) are also saved, and stomatal data are written into the csv.

(6) drop-down list “Camera_Settings”: Choose the image format, size and frame rate according to the camera connected.

(7) checkbox “Enable image analysis pipeline”: Apply the stomata detection program. When it is checked, the grayscale image (analysis input) and stomata-marked image (analysis output) are displayed.

(8) checkbox “Display Stream Logs”: Display the detected stomatal number.

(9) checkbox “Hide Input”: Switch ON/OFF of displaying the camera input which is an RGB color image. Regardless of whether it is checked or not, the camera input image will be saved after you press the “acquire image” button.

(10) The input grayscale image for the stomata detection program.

(11) The output image from the stomata detection program.

**Supplementary Figure 2. Stomatal file intervals.**

(A) The analysis flow to measure the interval lengths between stomatal files. Stomatal y-coordinates in SSD output (left most) are converted into stomatal density data (middle window) along y-axis dimension, then stomatal file positions are detected as peaks higher than a threshold. (B, C) Histogram of stomatal file intervals in the abaxial (B) and adaxial (C) surfaces. Green curves indicate density plots.

**Supplementary Figure 3. Predicted and hand-measured stomatal sizes.**

**(A) Stomatal length and width were defined as x- and y-lengths of the bounding box. (B) scatter** plot of the predicted (y-axis) and hand-measured (x-axis) stomatal lengths. (C) scatter plot of the predicted (y-axis) and hand-measured (x-axis) stomatal widths. The Pearson's correlation coefficients *r* are indicated in each plot window of B and C.

**Supplementary Figure 4. Effect of image resolution.**

Stomatal images of *Brachypodium distachyon* acquired with ×4 lens (upper) and ×10 lens (lower) are shown. scale bars: 100 μm.
